# Yorkie Negatively Regulates the Expression of Antimicrobial Proteins by Inducing Cactus Transcription in Prawns *Macrobrachium nipponense*

**DOI:** 10.3389/fimmu.2022.828271

**Published:** 2022-01-20

**Authors:** Ying Huang, Qin Si, Jie Du, Qian Ren

**Affiliations:** ^1^ Department of Marine Biology, College of Oceanography, Hohai University, Nanjing, China; ^2^ Biodiversity and Biosafety Research Center, Nanjing Institute of Environmental Sciences, Nanjing, China; ^3^ Animal Husbandry and Veterinary College, Jiangsu Vocational College of Agriculture and Forestry, Jurong, China; ^4^ College of Marine Science and Engineering, Nanjing Normal University, Nanjing, China

**Keywords:** *Macrobrachium nipponense*, yorkie, cactus, expression regulation, immune-related genes, innate immune response

## Abstract

The Hippo signaling pathway controls organ size and immune system in *Drosophila* and mammals. Yorkie acts as a transcriptional co-activator in the Hippo pathway and cross-talks with other essential pathways. In this study, a *Yorkie* gene and two *Cactus* isoforms (designated as *MnYorkie*, *MnCactus-a*, and *MnCactus-b*, respectively) were isolated and characterized from oriental river prawns (*Macrobrachium nipponense*). Results showed that *MnYorkie* includes 1620 bp open reading frame and encodes a protein of 539 amino acids (aa). *MnCactus-a* (377 aa) and *MnCactus-b* (471 aa) were produced by alternative splicing. *MnYorkie* and *MnCactus* were continuously expressed in all selected tissues. Upon Gram-positive bacterium *Staphylococcus aureus* and Gram-negative bacterium *Vibrio parahaemolyticus* stimulation, the mRNA levels of *MnYorkie* and *MnCactus* in hemocytes and intestines underwent time-dependent enhancement. RNA interference studies showed that *MnYorkie* silencing remarkably downregulated the transcription of *MnCactus* but upregulated the expression of seven immune-related genes. In addition, *MnYorkie* silencing *in vivo* decreased the susceptibility of prawns to bacterial challenge. After *S. aureus* and *V. parahaemolyticus* infection, the survival rate of prawns increased significantly from 2 to 6 days, which corresponded to the period of *MnYorkie* knockdown. All these findings suggested that *MnYorkie* in the Hippo pathway might exhibit remarkable biological roles in the immune defense of *M. nipponense* by negatively regulating the expression of immune-related genes and promoting the transcription of *MnCactus*.

## Introduction

Invertebrates, such as insects and crustaceans, rely mainly on various innate defense reactions to combat pathogen infections (1). When foreign pathogens invade, the innate immune system triggers various humoral and cellular activities through signal transduction pathways (2, 3). Signal transduction involves the binding of extracellular signaling molecular to cell–surface pattern recognition receptors (PRRs) to change the conformation of the receptor, a phenomenon also known as receptor activation (4). Some of the activated PRRs repress the growth or directly kill pathogens, and most of them transmit corresponding signals into the cell to trigger physiological responses (5). Several signaling pathways in invertebrates have been discovered, such as Janus kinase-signal transducer activator of transcription (JAK/STAT), immune deficiency (IMD), Toll, and Hippo pathways (6, 7).

The Hippo pathway was first identified as an evolutionarily conserved signaling mechanism that controls organ size by inhibiting cell proliferation and promoting apoptosis in *Drosophila* (8, 9). In canonical Hippo signaling, upstream stimuli activate the Ste-20 family protein kinase Hippo (Hpo), which then binds to and phosphorylates regulatory scaffold protein Salvador (Sav) (10). The Hpo/Sav complex and Mats (Mob as tumor suppressor) adaptor protein phosphorylate and activate Warts (Wts), a nuclear Dbf2-related family protein kinase (11). The Wts/Mats complex then phosphorylates the downstream target protein Yorkie (Yki), a transcriptional co-activator (12). Phosphorylated Yki is prohibited from entering the nucleus to interact with the transcription factor Scalloped (Sd), thus unable to trans-activate gene targets and is inhibited by Hippo signaling (12, 13). The components of *Drosophila* Hippo pathway are highly conserved in mammals. Hpo, Sav, Wts, Mats, and Yki in *Drosophila* are orthologs of mammalian Ste-20 kinases Mst1 and Mst2 (Mst), Sav, large tumor suppressor (Lats) 1/2, Mps one binder 1A and 1B (MOB), and Yes-associated protein (YAP), respectively (14, 15). Human Mst2, Lastl/2, MOB1, and YAP can rescue the corresponding *Drosophila* mutants and thus are functionally conserved in mammals (16).

In addition to its role in developing or regenerating tissues, the Hippo pathway regulates the immune system in *Drosophila* and mammals (17). Its dysregulation has been linked to various human cancers (15, 18). In *Drosophila*, the antibacterial response triggered by Hippo signaling can be enhanced by reducing the expression of Cactus, a Yki target gene (19) and an inhibitor of NF-κB (IκB) in the Toll pathway (20). In the absence of Hippo function, Gram-positive bacteria and fungi increase the lethality of flies. Without inhibition by Hpo, Yki translocates into the nucleus to upregulate Cactus transcription, which normally inhibits the nuclear translocation of NF-κB transcription factors Dorsal and Dorsal-related immunity factor (Dif) and the transcription of antimicrobial peptides (AMPs). Upon activation by Gram-positive bacteria, the Toll–Myd88–Pelle cascade leads to the phosphorylation and degradation of the Cka subunit of the Hippo inhibitory complex, thus eventually releasing Hpo to achieve Yki blockage and antimicrobial effects. Therefore, Hippo signaling enhances NF-κB signaling and AMP expression in *Drosophila* (21). Most of its components in *Drosophila* have been cloned in prawns, thereby suggesting the existence of Hippo pathway in these invertebrates. Nevertheless, the role of the Hippo pathway in immune regulation in prawns remains unknown.

As a member of the Palaemonidae family of decapod crustaceans, *Macrobrachium nipponense* is widely distributed in the freshwater and low-salinity estuarine regions of China, Japan, and South–East Asian countries (22). Prawns live in an aquatic environment full of bacteria, fungi, and other potential pathogens. Understanding the molecular mechanism of their innate immunity will contribute to disease control in prawn aquaculture. In this study, a *Yorkie* gene and two *Cactus* isoforms (named *MnYorkie*, *MnCactus-a*, and *MnCactus-b*, respectively) were identified from *M. nipponense*. Their tissue distributions and temporal response to bacterial stimulation were examined. The biological function of *MnYorkie* was investigated by detecting the expression of downstream immune-related genes, bacterial clearance activity, and organism survival rate. The findings will provide insights into the immune defense mechanisms of crustaceans.

## Materials and Methods

### Experimental Animals

A total of 200 healthy *M. nipponense* specimens with a weight of 2.5–3.5 g were purchased from an aquatic product market in Nanjing, China and kept in an aerated water tank filled with freshwater. After 10 days of acclimatization, hemolymph was extracted from five prawns and placed in an equal volume of an anticoagulant solution (glucose, 1.47 g; citric acid, 0.48 g; trisodium citrate, 1.32 g; prepared in ddH_2_O and added to 100 mL, pH 7.3). Hemocytes were then isolated through centrifugation at 2000 rpm for 10 min. Heart, hepatopancreas, gills, intestine, and stomach were dissected from the prawns and quickly stored at –80°C until RNA extraction.

### Bacterial Challenge and Tissue Collection

Seventy-five prawns were randomly selected for bacterial infections (23). For immune challenge, each prawn was injected with 50 μL of *S. aureus* (3 × 10^7^ cells) and *V. parahaemolyticus* (3 × 10^7^ cells) in phosphate-buffered saline (PBS,140 mM NaCl, 2.7 mM KCl, 10 mM Na_2_HPO_4_, 2 mM KH_2_PO_4_, pH 7.4). Hemocytes and intestines were collected from five live individuals at each time point (0, 12, 24, 36, and 48 h after injection) for total RNA extraction.

### Total RNA Extraction and cDNA Synthesis

Total RNA was isolated from the collected samples using the TRIzol Reagent (Invitrogen, USA) in accordance with the manufacturer’s protocols. RNA quality was assessed by electrophoresis on 1.5% agarose gel, and RNA concentration was measured by a NanoDrop 2000 Spectrophotometer (Thermo Scientific, USA). In brief, 1 μg of RNA was used to synthesize first-strand cDNA using the PrimeScript^®^ 1st Strand cDNA Synthesis Kit (Takara, Japan) with an oligo-dT primer. The obtained cDNA was kept at –20°C.

### Cloning of MnYorkie and MnCactus cDNAs

On the basis of the unigenes obtained from *M. nipponense* transcriptome data, four specific primers (*MnYorkie*-F: 5′-ATTGCGGATGAGTCTACGGGAGGAGGCTGG-3′ and *MnYorkie*-R: 5′-GACCAGCCTCCTCCCGTAGACTCATCC-3′; *MnCactus*-F: 5′-AACGGAGCCACGGGAATAGCCAATCTT-3′ and *MnCactus*-R: 5′-ATCATCTGCTGTGAGGTAGGCTGGGAGGGC-3′) were designed to clone the full-lengths of *MnYorkie* and *MnCactus*. A SMARTer™ RACE cDNA Amplification Kit (Clontech, USA) was used to synthesize the first-strand cDNA. The 5′- and 3′-rapid amplification of cDNA ends (RACE) was performed using an Advantage^®^ 2 PCR Kit (Clontech, USA) under the following PCR procedures: 94°C for 30 s, 70°C for 30 s, and 72°C for 3 min, followed by 20 cycles at 94°C for 30 s, 68°C for 30 s, and 72°C for 3 min. The PCR products were purified using a gel extraction kit (Takara, Japan) and sequenced (Springen, China) after insertion into the pEasy-T3 vector (TransGen Biotech, China).

### Sequence Analysis

Homology analysis was accomplished using the online BLAST algorithm at NCBI website (http://blast.ncbi.nlm.nih.gov/Blast.cgi). The deduced amino acid sequence was obtained using an open reading frame (ORF) finder program (https://www.ncbi.nlm.nih.gov/orffinder/) and then analyzed with the ExPASy Translate tool (https://web.expasy.org/translate/). Putative domains and motifs were predicted by the SMART program (http://smart.embl-heidelberg.de/). Molecular weight (Mw) and theoretical isoelectric point (pI) were calculated with the ExPASy Compute pI/Mw tool (https://web.expasy.org/compute_pi/). Multiple sequence alignments were carried out with the Clustal Omega program (https://www.ebi.ac.uk/Tools/msa/clustalo/) and GENEDOC software. A phylogenetic tree was constructed with MEGA 7.0 software using a neighbor-joining (NJ) method (24). Nodal support was assessed by 1000 bootstraps.

### Quantitative Real-Time PCR (RT-qPCR)

Two pairs of primers (*MnYorkie*-qF: 5′-GTGGTGGTGGTGGGTTTAG-3′ and *MnYorkie*-qR: 5′-GCAGAGATGCTGGTGAAGAA-3′; *MnCactus*-qF: 5′-CCACGGGAATAGCCAATCTT-3′ and *MnCactus*-qR: 5′-TCCAGTGACCTCATCGTAGT-3′) were synthesized to examine the tissue distribution and expression profiles of *MnYorkie* and *MnCactus* by RT-qPCR using the TransStart^®^ Top Green qPCR SuperMix Kit (TransGen Biotech, China) in LightCycler^®^ 96 real-time PCR Detection System (Roche, USA) under the following cycling conditions: 95°C for 30 s, 40 cycles of 95°C for 5 s, 60°C for 20 s, and a melting curve analysis from 65°C to 95°C. *β-actin* gene from *M. nipponense* was used as an internal PCR and cDNA template control and was positively amplified from all samples with primers (*β-actin*-qF: 5′-TATGCACTTCCTCACGCCATC-3′ and *β-actin*-qR: 5′-AGGAGGCGGCAGTGGTCAT-3′). All the experiments were repeated three times, and the obtained data were calculated using comparative CT (2^−ΔΔCt^) method (25). Statistical analysis was performed using unpaired sample *t*-test, and the level of significant difference was set at *P* < 0.05.

### Semi-Quantitative Reverse Transcription-PCR (SqRT-PCR)

The tissue distribution of *MnYorkie* and *MnCactus* was also detected by SqRT-PCR using specific primers (*MnYorkie*-SqF: 5′-CCTCCAATCCACTGCCTTATT-3′ and *MnYorkie*-SqR: 5′-GCTGAGGAGCCGAAGTTAAA-3′; *MnCactus*-SqF: 5′-AATGCAAGGGAAGGGAAGAG-3′ and *MnCactus*-SqR: 5′-GTCATCATCTGCTGTGAGGTAG-3′) under the following conditions: 94°C for 3 min, 30 cycles of 94°C for 30 s, 53°C for 45 s, and 72°C for 30 s, and 72°C for 5 min. *β-actin* was amplified as reference gene for internal standardization (*β-actin*-SqF: 5′-AATGTGTGACGACGAAGTAG-3′ and *β-actin*-SqR: 5′-GCCTCATCACCGACATAA-3′). The PCR products were separated on agarose gels and photographed over NV light using Quantity One software (Bio-Rad, Hercules, CA).

### RNA Interference (RNAi) of MnYorkie *In Vivo*

Primers specific to *MnYorkie* (*MnYorkie*-dsRNA-F: 5′-GCGTAATACGACTCACTATAGGGGTAGTGACCTGGTGTTG-3′ and *MnYorkie*-dsRNA-R: 5′-GCGTAATACGACTCACTATAGGCGCAGGCAAATGTCTCTTC-3′) and green fluorescent protein (GFP-dsRNA-F: 5′-GCGTAATACGACTCACTATAGGTGGTCCCAATTCTCGTGGAAC-3′ and GFP-dsRNA-R: 5′-GCGTAATACGACTCACTATAGGCTTGAAGTTGACCTTGATGCC-3′) were designed to synthesize DNA template for the transcription of *MnYorkie*-dsRNA and GFP-dsRNA. Double stranded RNAs (dsRNA) were prepared *in vitro* by using the HiScribe™ T7 quick high-yield RNA synthesis kit (BioLabs, USA). The prawns were initially injected with 50 μL of PBS containing 15 μg of *MnYorkie*-dsRNA or GFP-dsRNA (as control). After 12 h, 15 μg of *MnYorkie*-dsRNA or GFP-dsRNA was injected into the same prawn. At 36 h post-injection (hpi), the RNAi efficiency of *MnYorkie* in hemocytes and intestines was checked using RT-qPCR.

### Measuring the Expression of Immune-Related Genes After MnYorkie Knockdown

At 36 h post *MnYorkie*-dsRNA or GFP-dsRNA injection, the prawns were further injected with *S. aureus* or *V. parahaemolyticus* (3 × 10^7^ cells) for immune challenge experiments. At 24 h post *S. aureus* or *V. parahaemolyticus* infection, the hemocytes and intestines were sampled, and the expression levels of several immune-related genes, namely, *MnCactus*, anti-lipopolysaccharide factor 1 (*MnALF1*), *MnALF2*, *MnALF3*, crustin 4 (*MnCrus4*), *MnCrus5*, *MnCrus6*, and lysozyme (*MnLyso1*) were determined using RT-qPCR with primers (*MnALF1*-qF: 5′-GTGGTGCCCAGGATGGACTT-3′ and *MnALF1*-qR: 5′-AGAGGATGGTGGAGGAAATT-3′; *MnALF2*-qF: 5′-AGAACCACCTGAACCCAACG-3′ and *MnALF2*-qR: 5′-TGACAGATTAAGCCAGCCCC-3′; *MnALF3*-qF: 5′-GTCGATGGAGTGTATGATGAGG-3′ and *MnALF3*-qR: 5′-GTAGTGCAGCTCGAGTCTTT-3′; *MnCrus4*-qF: 5′-GGAATTAGAAGGGCCCGTCGG-3′ and *MnCrus4*-qR: 5′-TCATAGCAGCACTTGTCAGCG-3′; *MnCrus5*-qF: 5′-ACACCCCAATCACCCCCCA-3′ and *MnCrus5*-qR: 5′-TGCCTTGAAACGGCTCCCT-3′; *MnCrus6*-qF: 5′-CTCCGTGTCCTCCCATACC-3′ and *MnCrus6*-qR: 5′-AGTTCCCTGTCGACTTCCT-3′; *MnLyso1*-qF: 5′- GGCAGAGGCAAGAGTTGATTA-3′ and *MnLyso1*-qR: 5′-GTTAGGATTCTCGTCCGATGTTAG-3′). Three independent experiments were performed in triplicate. The results were subjected to one-way ANOVA using SPSS 19.0, and *P* value less than 0.05 was considered statistically significant.

### Assay of Bacterial Clearance

In brief, 30 μg of *MnYorkie*-dsRNA or GFP-dsRNA was injected into the prawns twice. At 24 h post second dsRNA injection, 50 μL of *S. aureus* or *V. parahaemolyticus* (3 × 10^7^ cells) was injected into the prawns. The hemolymph from 10 prawns was extracted and mixed with anticoagulant buffer at 20 min post-bacterial injection. After dilution 1:100/1:1000 with PBS, 50 μL of the hemolymph was loaded on LB agar plates and incubated at 37°C overnight. Colony forming unit (cfu) in the plate was counted. The number of *S. aureus* or *V. parahaemolyticus* in prawn hemolymphs was calculated and expressed as cfu mL^−1^ hemolymph. The assay was repeated in triplicate for all samples.

### Prawn Survival Rate

Eighty prawns were divided into four groups (*MnYorkie*-dsRNA plus *S. aureus* group, GFP-dsRNA plus *S. aureus* group, *MnYorkie*-dsRNA plus *V. parahaemolyticus* group, and GFP-dsRNA plus *V. parahaemolyticus* group) to determine whether *MnYorkie* is involved in its host’s antibacterial immune defense. Twenty prawns per each experimental group were injected with 30 μg of *MnYorkie*-dsRNA or GFP-dsRNA twice and then with 50 μL of *S. aureus* (3 × 10^7^ cells) or *V. parahaemolyticus* (3 × 10^7^ cells) at 24 h after the second injection. The number of dead prawns in these four treatment groups was monitored, and the cumulative survival rate of prawns was calculated and recorded up to 144 h. The experiment was repeated three times.

## Results

### Sequence Characters and Phylogenetic Analysis of MnYorkie

**Figure 1:**
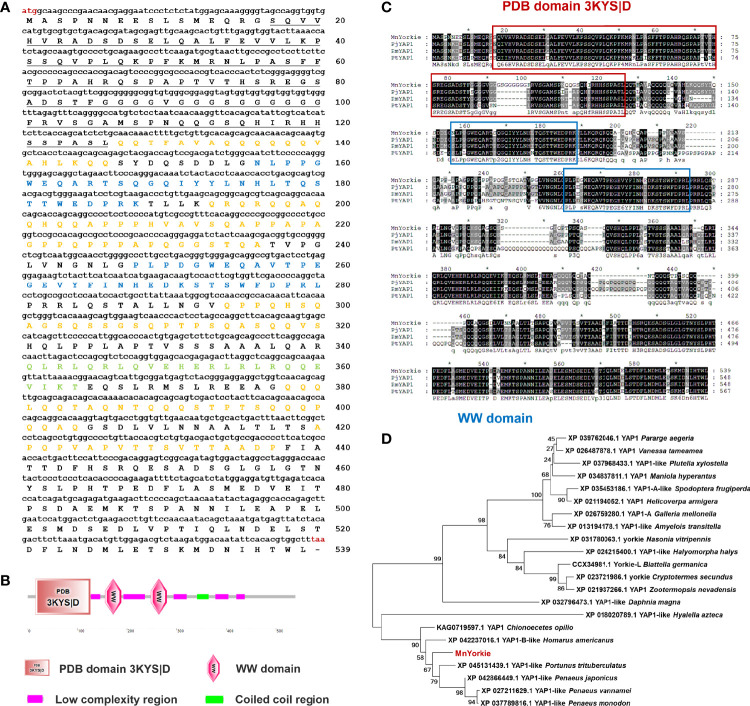
**(A)** Nucleotide (upper case) and deduced amino acid (lower case) sequence of MnYorkie from *M. nipponense.* The sequences were shown and numbered along the right margin. The red letters indicated the start codon (ATG) and the stop codon (TAA). The PDB domain 3KYS|D was underlined. Two WW domains were shown in blue. The coiled coil region and five low complexity regions were marked in green and orange, respectively. **(B)** Domain organization of MnYorkie predicted by SMART. **(C)** Multiple-sequence alignment of MnYorkie with other crustacean YAPs. The identical amino acid residues were shaded in black and the similar residues in gray. The sequences used for the multiple sequence alignment included PjYAP1 from *P. japonicus* (XP_042866449.1); PmYAP1 from *P. monodon* (XP_037789816.1) and PtYAP1 from *P. trituberculatus* (XP_045131439.1). **(D)** Phylogenetic tree of YAPs. The alignment was created using MEGA 7.0 based on the amino acid sequences of 21 YAP family members from different organisms. The phylogenetic tree was built by the NJ algorithm, and the numbers at the nodes indicated the bootstrap value. MnYorkie was shown in red.

The full-length *MnYorkie* mRNA (GenBank Accession No. OL901207) is 2558 bp long consisting of a 75 bp 5′-untranslated region (UTR), an 863 bp 3′-UTR, and a 1620 bp ORF encoding a predicted polypeptide of 539 amino acids with a calculated Mw of 58.3 kDa and a pI of 5.29 (**Figure 1A**). Conserved domain analysis revealed that MnYorkie contains a PDB domain 3KYS|D of 110 amino acids located at 16–126 in the N-terminal region, two WW (domain with 2 conserved Trp (W) residues) domains located at 156–188 and 248–280, five low complexity regions located at 127–146, 193–236, 294–320, 378–404 and 420–437, and a coiled coil region of 24 amino acids located at 341–364 (**Figure 1B**).

The deduced amino acid sequence of MnYorkie was aligned with YAP family members as shown in **Figure 1C**. MnYorkie showed 75.22% identity to PjYAP1 from *Penaeus japonicus*; 72.01% to PmYAP1 from *Penaeus monodon*, and 68.85% to PtYAP1 from *Portunus trituberculatus*. A phylogenetic tree was constructed based on the amino acid sequences of 22 YAP members using NJ method to evaluate its evolutionary position. MnYorkie was nearly clustered with crustacean YAPs, namely, PtYAP1, PjYAP1, PvYAP1, PmYAP1, HaYAP1-B, and CoYAP1 (**Figure 1D**).

### Cloning and Characterization of MnCactus

**Figure 2:**
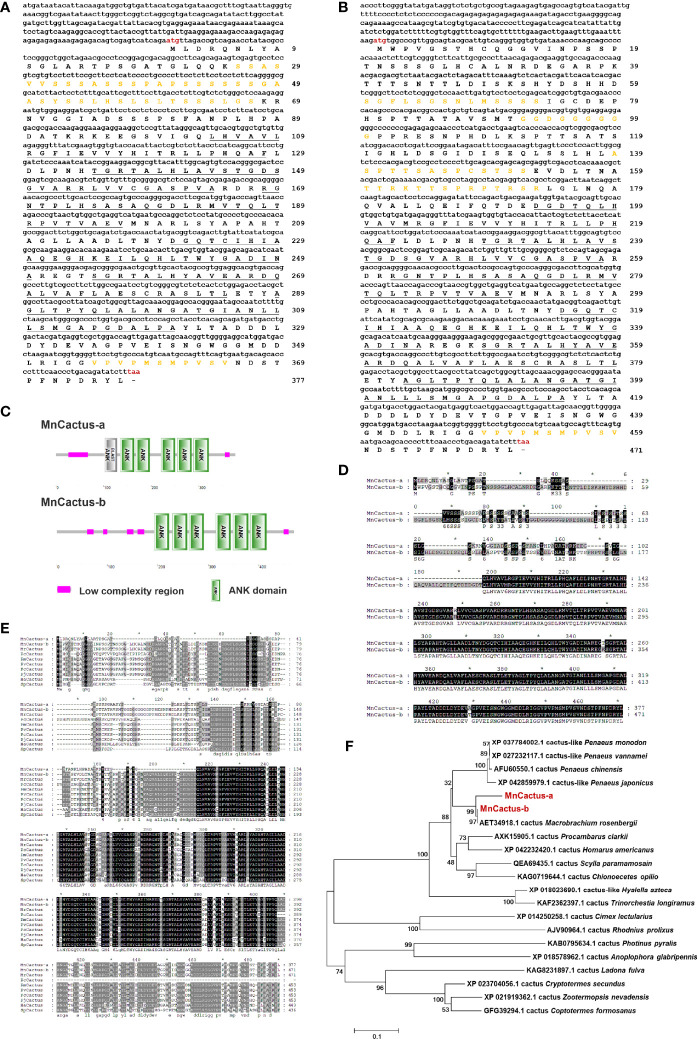
Nucleotide and deduced amino acid sequences of MnCactus-a **(A)** and MnCactus-b **(B)** from *M. nipponense.* The sequences were shown and numbered along the right margin. The red letters indicated the start codon (ATG) and the stop codon (TAA). The ANK domains were underlined, and the low complexity regions were marked in orange. **(C)** Domain organizations of MnCactus-a and MnCactus-b predicted by SMART. **(D)** The sequence alignment of MnCactus-a and MnCactus-b. **(E)** Multiple sequence alignment of MnCactus with other Cactus sequences. The identical amino acid residues were shaded in black and the similar residues in gray. The species and the GenBank accession numbers were as follows: MrCactus from *M. rosenbergii* (AET34918.1); PcCactus from *P. clarkii* (AXK15905.1); PmCactus from *P. monodon* (XP_037784002.1); PvCactus from *P. vannamei* (XP_027232117.1); FcCactus from *P. chinensis* (AFU60550.1); PjCactus from *P. japonicus* (XP_042859979.1); HaCactus from *H. americanus* (XP_042232420.1); SpCactus from *S. paramamosain* (QEA69435.1). **(F)** NJ phylogenetic tree analysis of the amino acid sequences of 21 Cactus from different species. Bootstrap resampling (1000 pseudo-replicates) was used to test the reliability of the branching. MnCactus-a and MnCactus-b were marked in red.

Two contigs of *MnCactus* were identified from *M. nipponense* transcriptome and referred to as *MnCactus-a* (GenBank Accession No. OL901208) and *MnCactus-b* (GenBank Accession No. OL901209) based on their nucleotide sequences. *MnCactus-a* transcript is 1614 bp long and consists of a 275 bp 5′-UTR, a 205 bp 3′-UTR, and a 1134 bp ORF encoding a 377 amino acid protein (**Figure 2A**). The theoretical pI and Mw of MnCactus-a protein are 5.98 and 39.34 kDa, respectively. SMART analysis showed that MnCactus-a contains six ankyrin (ANK) domains located at 103–128, 135–164, 168–197, 221–250, 255–285, and 289–321 and two low complexity regions located at 26–67 and 355–365. The obtained *MnCactus-b* cDNA sequence is 1866 bp long with a 245 bp 5′-UTR, a 205 bp 3′-UTR, and a 1416 bp ORF that encodes a 471 amino acid protein with a Mw of 49.5 kDa and a pI of 5.45 (**Figure 2B**). MnCactus-b contains six ANK domains located at 193–222, 229–258, 262–291, 315–344, 347–379, and 383–415 and five low complexity regions located at 60–73, 92–100, 139–152, 160–173, and 449–459 (**Figures 2C, D**).

The deduced amino acid sequences of MnCactus-a and MnCactus-b shared high sequence similarities to Cactus from other species. MnCactus-a and MnCactus-b showed 95.44% and 98.73% identity to MrCactus from *Macrobrachium rosenbergii*, respectively; 83.74% and 65.58% to PcCactus from *Procambarus clarkii*, respectively; 80.7% and 69.11% to PmCactus from *P. monodon*, respectively; 80.7% and 67.64% to PvCactus from *P. vannamei*, respectively; 80.35% and 68.99% to FcCactus from *Penaeus chinensis*, respectively; 79.65% and 68.79% to PjCactus from *P. japonicus*, respectively; 78.95% and 67.94% to HaCactus from *H. americanus*, respectively; 74.48% and 66.75% to SpCactus from *Scylla paramamosain*, respectively; and 63.61% and 63.95% to CoCactus from *C. opilio*, respectively (**Figure 2E**). In the phylogenetic tree, MnCactus-a and MnCactus-b were first clustered with MrCactus; then grouped with four shrimp Cactus including PjCactus, PcCactus, PvCactus, and PmCactus; and finally clustered into crustacean Cactus including PcCactus, HaCactus, SpCactus, and CoCactus (**Figure 2F**).

### Tissue Distributions of MnYorkie and MnCactus

**Figure 3:**
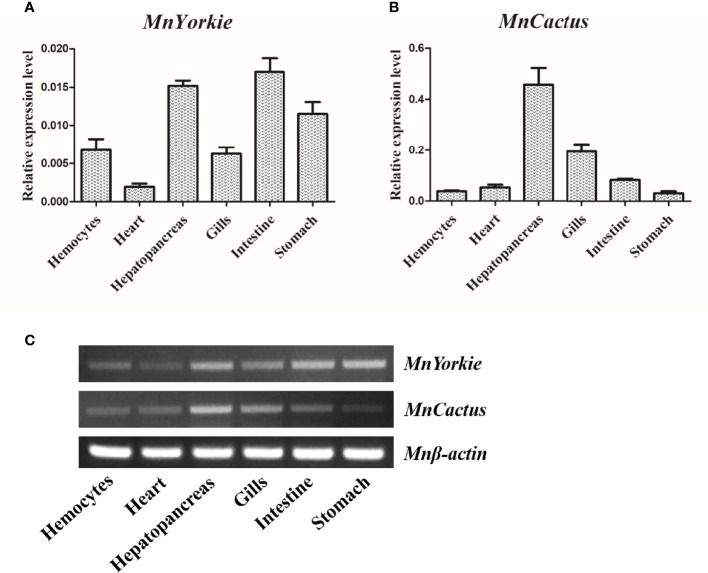
Tissue distributions of *MnYorkie* and *MnCactus* in *M. nipponense.* The relative expression levels of *MnYorkie* and *MnCactus* in different tissues (hemocytes, heart, hepatopancreas, gills, intestines and stomach) were measured by RT-qPCR **(A, B)** and SqRT-PCR **(C)**. *β-actin* was used as internal control in all tissues. The values were shown as mean ± SD, N = 5.

The expression levels of *MnYorkie* and *MnCactus* in normal *M. nipponense* tissues were detected by RT-qPCR (**Figures 3A, B**) and SqRT-PCR (**Figure 3C**). The single peak on the dissociation curve showed the specific amplifications of *MnYorkie*, *MnCactus*, and *β-actin*. *MnYorkie* expression was highest in the intestines; relatively high in hepatopancreas, stomach, hemocytes, and gills, and the lowest in the heart. *MnCactus* mRNA transcript was constitutively expressed in all selected tissues with high expression level in hepatopancreas and gills.

### Expression Profiles of MnYorkie and MnCactus Upon Bacterial Challenge

**Figure 4:**
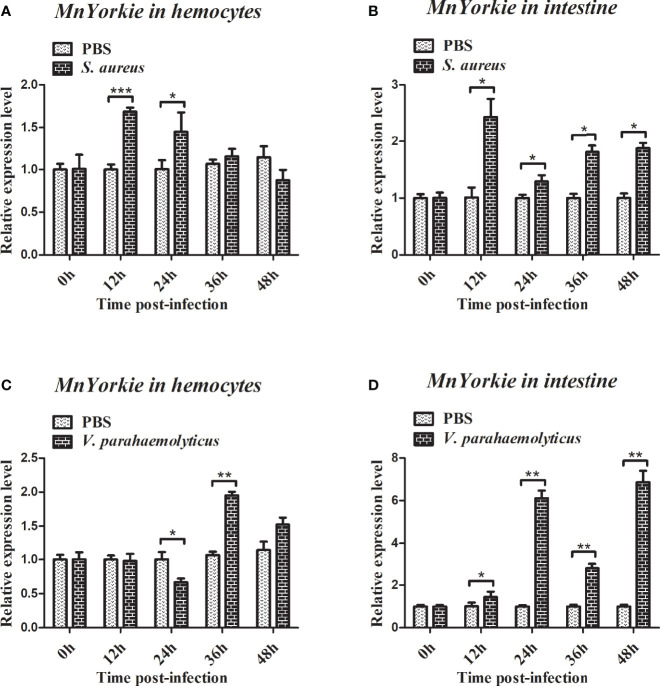
Relative transcription levels of *MnYorkie* in the hemocytes and intestines of bacterial-infected prawns. At 0, 12, 24, 36, and 48 h after *S. aureus* **(A, B)** or *V. parahaemolyticus* **(C, D)** infection in prawns, hemocytes and intestines were collected and processed for the RT-qPCR analysis of *MnYorkie* expression. *β-actin* was used as an internal control. Data are presented as mean ± SD, N = 5. Significance was compared between the experimental group and the PBS-injected group at the same time point. Significant differences are indicated with an asterisk at *P* < 0.05, two asterisks at *P* < 0.01 or three asterisks at *P* < 0.001.

**Figure 5:**
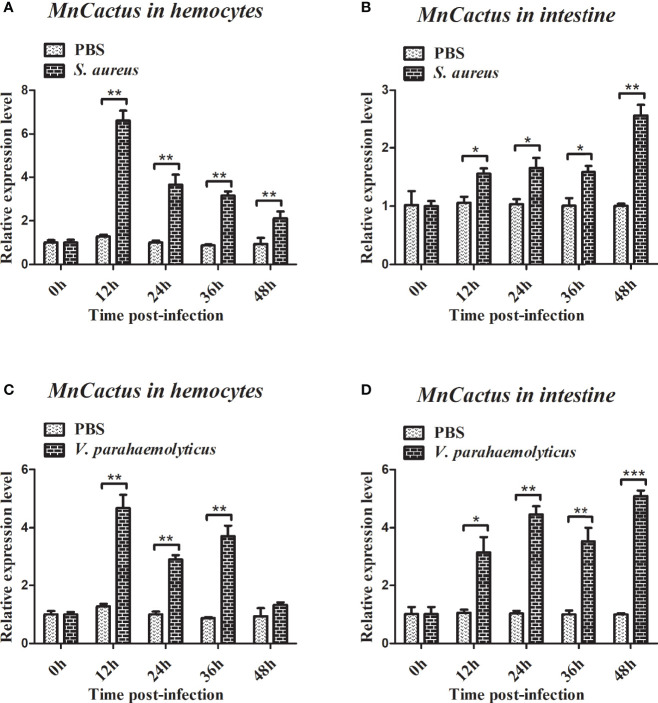
Relative transcription levels of *MnCactus* in the hemocytes and intestines of bacterial-infected prawns. At 0, 12, 24, 36, and 48 h after *S. aureus* **(A, B)** or *V. parahaemolyticus* **(C, D)** infection in prawns, hemocytes and intestines were collected and processed for the RT-qPCR analysis of *MnCactus* expression. *β-actin* was used as an internal control. Data are presented as mean ± SD, N = 5. Significance was compared between the experimental group and the PBS-injected group at the same time point. Significant differences are indicated with an asterisk at *P* < 0.05, two asterisks at *P* < 0.01 or three asterisks at *P* < 0.001.

RT-qPCR was used to detect the temporal mRNA expression of *MnYorkie* and *MnCactus* in hemocytes and intestines after *S. aureus* and *V. parahaemolyticus* challenge. Upon *S. aureus* challenge, the expression level of *MnYorkie* in hemocytes was greatly upregulated at 12 hpi and recovered to the control level from 24 hpi to 48 hpi (**Figure 4A**); *MnYorkie* mRNA in intestines was dramatically upregulated at 12 hpi, then downregulated at 24 hpi, and increased from 36 hpi to 48 hpi (**Figure 4B**); Upon *V. parahaemolyticus* challenge, the transcript of *MnYorkie* in hemocytes decreased at 24 h and then increased to the highest level at 36 hpi (**Figure 4C**); Its expression in intestines sharply increased to the first peak at 24 hpi, then decreased at 36 hpi, and reached the maximum level at 48 hpi (**Figure 4D**). After *S. aureus* challenge, the expression of *MnCactus* in hemocytes increased to its respective highest level at 12 hpi and was then slightly downregulated from 24 hpi to 48 hpi (**Figure 5A**); With time, the expression level of *MnCactus* mRNA in intestines increased gradually and reached the highest level at 48 hpi (**Figure 5B**); After *V. parahaemolyticus* infection, the transcript of *MnCactus* increased sharply at 12 hpi, then decreased slightly at 24 hpi, increased again at 36 hpi, and returned to normal levels at 48 hpi (**Figure 5C**); *MnCactus* expression in intestines continued to upregulate from 12 hpi to 48 hpi (**Figure 5D**). These results revealed the potential role of *MnYorkie* and *MnCactus* in prawn immunity.

### Gene Knockdown

**Figure 6:**
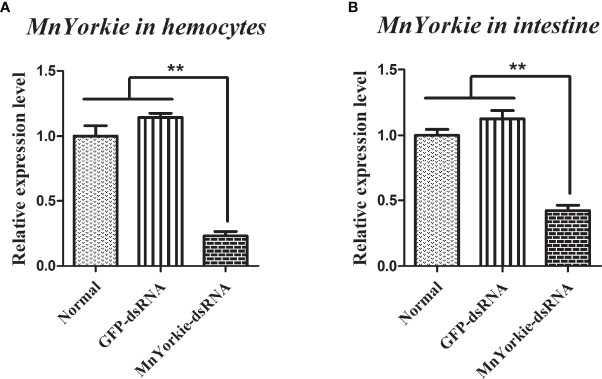
Analysis of *MnYorkie* expression after *MnYorkie* RNAi. mRNA expression of *MnYorkie* in hemocytes **(A)** and intestines **(B)** after injection with *MnYorkie*-dsRNA 36 h were determined by RT-qPCR. All data were normalized to GFP-dsRNA treated samples and *β-actin* was used as an internal reference. Data are presented as mean ± SD, N=5. The asterisk indicated significant difference between *MnYorkie*-dsRNA group and GFP-dsRNA group (**P < 0.01).

*MnYorkie*-dsRNA was synthesized successfully and used to challenge the prawns to further explore the function of *MnYorkie in vivo*. The relative expression levels of *MnYorkie* in hemocytes and intestines after *MnYorkie*-dsRNA interference are shown in in **Figures 6A, B**, respectively. The most significant effect of *MnYorkie* was detected at 36 hpi. The silencing efficiency of *MnYorkie*-dsRNA reached up to 76.88% in hemocytes and 57.72% in intestines. Thus, 36 h after dsRNA injection was selected as the optimum time of *MnYorkie* knockdown and used for further RNAi experiments.

### Effects of MnYorkie Knockdown on the Expression of MnCactus and Immune-Related Genes

**Figure 7:**
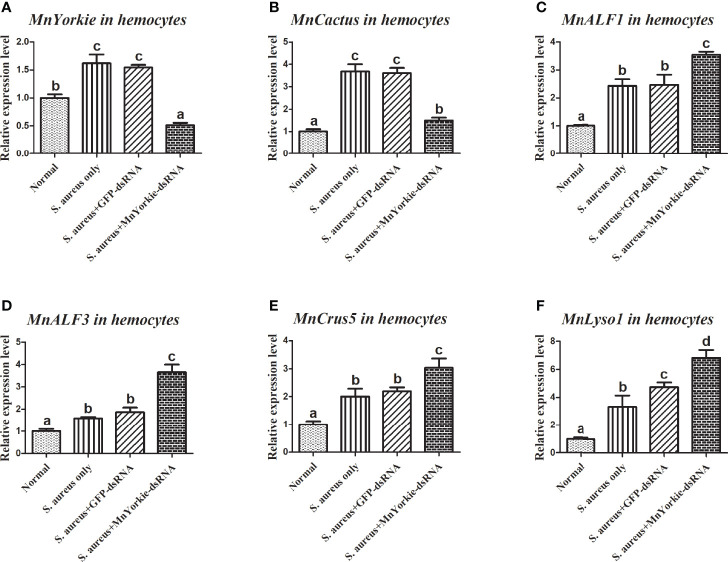
Analysis of *MnCactus* and four immune-related genes expression levels after *MnYorkie* RNAi. **(A)** Efficiency of RNAi detected by RT-qPCR in hemocytes at 36 h post of *MnYorkie*-dsRNA and *S. aureus* injection. The mRNA expression levels of *MnCactus* **(B)**, *MnALF1* **(C)**, *MnALF3* **(D)**, *MnCrus5* **(E)**, *MnLyso1* **(F)** were analyzed by RT-qPCR at 24 h injection of *S. aureus* post *MnYorkie* RNAi. Significance differences were compared between the normal group to other treated groups at the same time point. Significant differences were marked with different letters (a, b, c, d).

**Figure 8:**
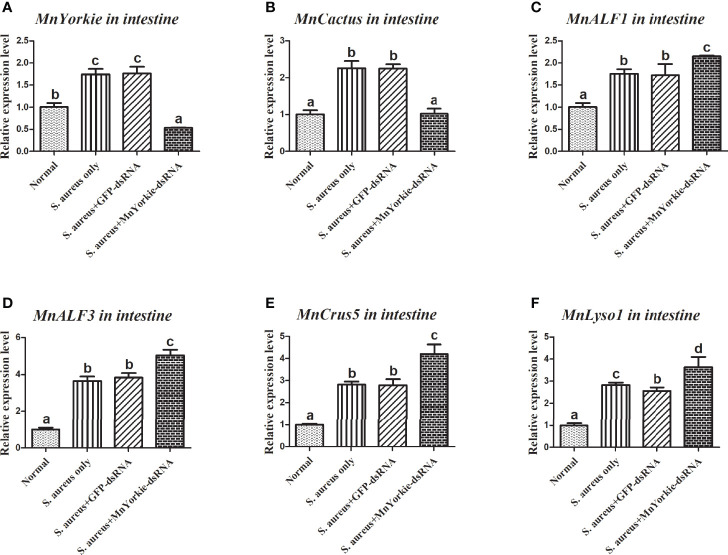
Analysis of *MnCactus* and four immune-related genes expression levels after *MnYorkie* RNAi. **(A)** Efficiency of RNAi detected by RT-qPCR in intestine at 36 h post of *MnYorkie*-dsRNA and *S. aureus* injection. The mRNA expression levels of *MnCactus* **(B)**, *MnALF1* **(C)**, *MnALF3* **(D)**, *MnCrus5* **(E)**, *MnLyso1* **(F)** were analyzed by RT-qPCR at 24 h injection of *S. aureus* post *MnYorkie* RNAi. Significance differences were compared between the normal group to other treated groups at the same time point. Significant differences were marked with different letters (a, b, c, d).

**Figure 9:**
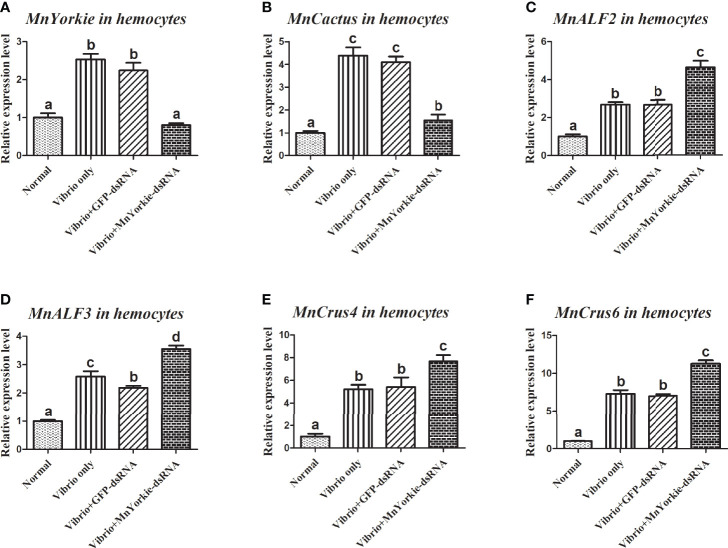
Analysis of *MnCactus* and four immune-related genes expression levels after *MnYorkie* RNAi. **(A)** Efficiency of RNAi detected by RT-qPCR in hemocytes at 36 h post of *MnYorkie*-dsRNA and *V. parahaemolyticus* injection. The mRNA expression levels of *MnCactus* **(B)**, *MnALF2* **(C)**, *MnALF3* **(D)**, *MnCrus4* **(E)**, *MnCrus6* **(F)** were detected by RT-qPCR at 24 h injection of *V. parahaemolyticus* post *MnYorkie* RNAi. Significance differences were compared between the normal group to other treated groups at the same time point. Significant differences were marked with different letters (a, b, c, d).

**Figure 10:**
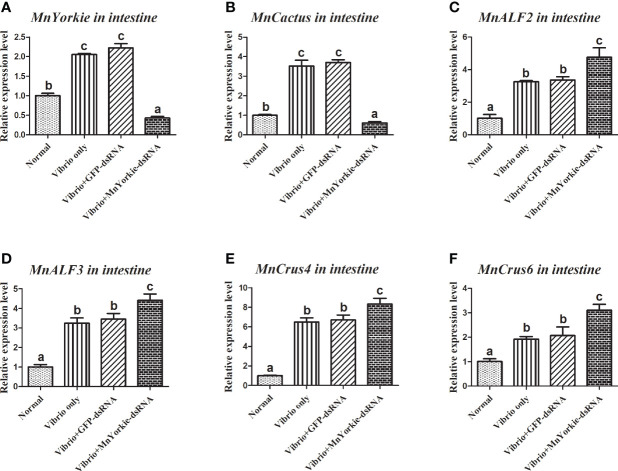
Analysis of *MnCactus* and four immune-related genes expression levels after *MnYorkie* RNAi. **(A)** Efficiency of RNAi detected by RT-qPCR in intestine at 36 h post of *MnYorkie*-dsRNA and *V. parahaemolyticus* injection. The mRNA expression levels of *MnCactus* **(B)**, *MnALF2* **(C)**, *MnALF3* **(D)**, *MnCrus4* **(E)**, *MnCrus6* **(F)** were detected by RT-qPCR at 24 h injection of *V. parahaemolyticus* post *MnYorkie* RNAi. Significance differences were compared between the normal group to other treated groups at the same time point. Significant differences were marked with different letters (a, b, c, d).

After the stimulations with *S. aureus* and *V. parahaemolyticus*, the mRNA transcripts of *MnCactus*, *MnALF1*, *MnALF2*, *MnALF3*, *MnCrus4*, *MnCrus5*, *MnCrus6*, and *MnLyso1* increased significantly at 24 hpi. Upon exposure to *S. aureus* for 24 h, the expression levels of *MnCactus* in hemocytes (**Figure 7B**) and intestines (**Figure 8B**) were greatly suppressed (*P* < 0.05) in the prawns with effective *MnYorkie* knockdown (**Figures 7A** and **8A**). On the contrary, four immune-related genes, namely, *MnALF1* (**Figures 7C** and **8C**), *MnALF3* (**Figures 7D** and **8D**), *MnCrus5* (**Figures 7E** and **8E**), *MnLyso1* (**Figures 7F** and **8F**) were upregulated (*P* < 0.05) in the *MnYorkie*-dsRNA injection group compared with those in the GFP-dsRNA group. No change was observed in the expression of *MnALF2*, *MnCrus4*, and *MnCrus6* (data not shown). Following *MnYorkie* knockdown and exposure to *V. parahaemolyticus* for 24 h (**Figures 9A** and **10A**), the expression levels of *MnCactus* in hemocytes (**Figure 9B**) and intestines (**Figure 10B**) were significantly suppressed (*P* < 0.05), and those of four *AMP* genes, *MnALF2* (**Figures 9C** and **10C**), *MnALF3* (**Figures 9D** and **10D**), *MnCrus4* (**Figures 9E** and **10E**) and *MnCrus6* (**Figures 9F** and **10F**) were remarkably upregulated (*P* < 0.05). These results suggested that *MnYorkie* negatively regulated the expression of several immune-related genes in *M. nipponense*.

### Bacterial Clearance Activity Upon MnYorkie Knockdown

**Figure 11:**
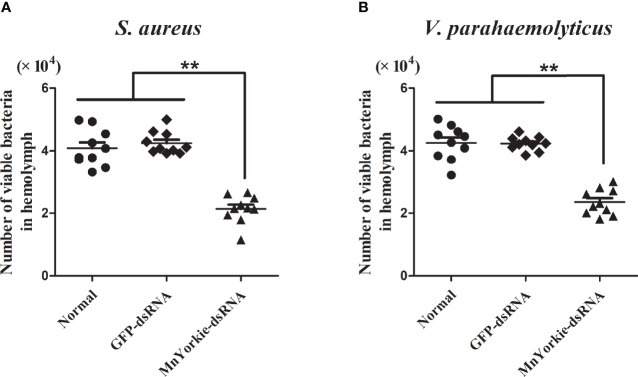
*In vivo* bacterial clearance assay. 36 h after *MnYorkie* RNAi, the number of bacterial colonies were counted and recorded with or without injected *S. aureus* **(A)** or *V. parahaemolyticus* **(B)** at 20 min. Data are shown as mean values ± S.D. (N = 10). The asterisks indicated significant differences (**P < 0.01).

The bacterial clearance activity of *MnYorkie* towards *S. aureus* (**Figure 11A**) and *V. parahaemolyticus* (**Figure 11B**) was tested to investigate the *in vivo* effects of *MnYorkie* on pathogen invasion. A gradual decrease in bacteria count was detected in all experimental groups. At 20 min post *S. aureus* or *V. parahaemolyticus* injection, the number of bacteria retained in the *MnYorkie*-dsRNA group was lower than that in the control group. This finding indicated a high elimination rate in the *MnYorkie*-dsRNA group. Meanwhile, no significant difference was detected between the GFP-dsRNA and control groups.

### MnYorkie Knockdown Promoted the Survival Rate of M. nipponense

**Figure 12:**
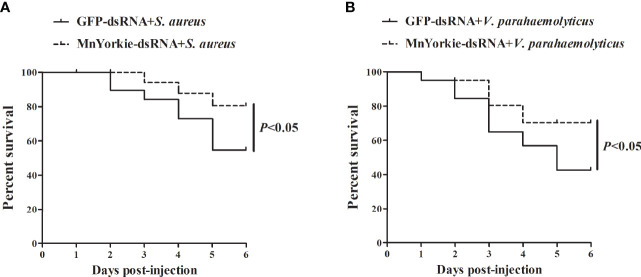
Evaluation of *M. nipponense* survival rate. The prawns were co-injected with *MnYorkie*-dsRNA and *S. aureus* **(A)** or *V. parahaemolyticus* **(B)**. At various times (0–6 days) after infection, the survival rate of prawn was examined.

Survival rate analysis was performed to evaluate the significance of *MnYorkie* to prawns during bacterial infections. The survival rate of prawns stimulated by *S. aureus* (**Figure 12A**) and *V. parahaemolyticus* (**Figure 12B**) presented different variation trends after *MnYorkie* knockdown. Compared with that of the GFP-dsRNA group, the survival rate of the *MnYorkie*-dsRNA plus *V. parahaemolyticus* or *S. aureus* group increased significantly after 2–6 days. These results suggested that *MnYorkie* silencing promoted the survival rate of prawns infected with bacteria and reduced their cumulative mortality.

## Discussion

The roles of Yorkie/YAP, a transcriptional co-activator protein, in Hippo signaling pathway of insects and mammals have been extensively studied. However, its function in the innate immunity of crustaceans remains to be elucidated. In this study, a *Yorkie* gene from *M. nipponense* (*MnYorkie*) was identified and functionally studied. Sequence structure and homologous phylogenetic analysis revealed that the predicted MnYorkie protein exhibits a classical Yorkie structure. Two 33 amino acid WW domains were found in MnYorkie. Given that one of the outstanding features of this sequence motif is the presence of two conserved tryptophans (W), it was named as the WW domain (26). These domains are small, conserved protein modules that are composed of approximately 40 amino acids (27), found in many different signaling and structural proteins, appears folded into a stable, triple stranded β-sheet, and recognized as proline-containing ligands (28). The same structural features indicated that MnYorkie has a potential transcriptional regulatory function in *M. nipponense*.

As a homolog of IκB, Cactus mediates negative- and positive-feedback regulatory loops of NF-κB *via* different pathways in invertebrates (29). In *Drosophila*, Cactus is a target gene of Yorkie in the Hippo pathway (19). Here, two Cactus isoforms (*MnCactus-a* and *MnCactus-b*) were cloned and identified from *M. nipponense* through transcriptomic analysis and amplification by PCR. The deduced MnCactus-a and MnCactus-b amino acid sequences contained the typical characteristics of IκB members: an N-terminal serine-rich region with conserved lysine and serine residues crucial for signal-dependent degradation (30) and a C-terminal ANK repeat domain (six motifs) that binds to the dimerization domain of NF-κB dimers (31). MnCactus-b also consists of a PEST motif (a region rich in proline, glutamate, serine, and threonine residues) at the N-terminus, which is required for the phosphorylation and intrinsic stability of IκB proteins (32). When a cell receives various stimulus signals, NF-κB is released from IκB and then quickly enters the nucleus to activate the expression of downstream effectors (33). Multiple sequence alignment analysis indicated that MnCactus-a and MnCactus-b showed sequence similarities to other crustaceans Cactus proteins. Full-length analysis using NJ method clustered these two into the shrimp group, suggesting their similar functions. These two MnCactus isoforms were formed by alternative splicing; however, the specific splicing method must be further studied in depth.

The tissue localization of *MnYorkie* transcripts showed its wide expression in all selected tissues and its abundance in intestines and hepatopancreas. *Cactus* is widely distributed but has distinguished expression profiles among different animals. In white shrimp *Litopenaeus vannamei*, *LvCactus* is highly expressed in the heart and muscles but lowly expressed in hepatopancreas (34). *FcCactus* is highly expressed in the muscles and hemocytes of Chinese shrimp *Fenneropenaeus chinensis* (35). In *M. nipponense*, *MnCactus* mRNA was highly expressed in hepatopancreas, the center of lipid and carbohydrate metabolism with a crucial role in removing potential microbial pathogens (36). The abundance of *MnYorkie* and *MnCactus* in hepatopancreas indicated their potential immune defense functions in *M. nipponense*.

In *Drosophila*, Hippo signaling is acutely activated only by Gram-positive bacteria (19). In this study, the Hippo pathway in *M. nipponense* was activated by Gram-positive and -negative bacteria. The mRNA levels of *MnYorkie* and *MnCactus* in hemocytes and intestines underwent time-dependent enhancement following bacterial stimulation. These results are not entirely new; similar expression patterns of Cactus have been reported in *L. vannamei* following lipopolysaccharides, poly (I:C), *V. parahaemolyticus* and *S. aureus* injections (34) and in *F. chinensis* after *Micrococcus lysodeikticus* and *Vibrio anguillarium* infections (35). The significant increase in the production of *MnYorkie* and *MnCactus* indicated their involvement in the immune response induced by bacteria. In other crustaceans, such as shrimp, lobsters, crayfish, and crabs, the immune response induced by bacteria could be regulated by various signaling pathways (29). The activity and expression of *MnYorkie* and *MnCactus* could be directly or indirectly regulated by feedback loops or other signaling pathways to achieve the homeostasis of immune responses.

Signal transduction comprises cascade amplification, dispersion, and regulation. One signaling molecule can activate the transduction of multiple signal pathways. Different combinations of several signals may also cause different responses, and coordinated interactions might occur between signal pathways (37). In *Drosophila* intestinal stem cells, the Hippo pathway is necessary for stem cell division in response to tissue damage *via* JAK/STAT and epidermal growth factor receptor signaling pathways (Ren et al., 2010). By inhibiting the activity of the Notch pathway, Yorkie acts as an important repressor in specifying polar cells during oogenesis in *Drosophila* (38). Therefore, the cross regulation of the Hippo pathway and other essential pathways may also exist in other invertebrate species. In previous research, the *MnHippo* gene in *M. nipponense* has been identified and subjected to functional analysis (23). Similar to *D. melanogaster* Hippo, *MnHippo* knockdown significantly reduced the expression of multiple immune-associated genes, such as *MnALF1*, *MnALF4*, *MnCrus5*, *MnCrus7*, and *MnLyso2* (23). Another immune protein in the Hippo pathway of *M. nipponense*, MnMOB1, has also been cloned and characterized. MnMOB1 has a positive effect on the expression of AMPs secreted into the extracellular space to resist microbial invaders (39). AMPs are a group of molecules with Mw usually less than 10 kDa and are the major antibacterial effectors in humoral immunity (29). In crustaceans, AMPs are critical immune responsive genes in the NF-κB pathways, including Toll and IMD pathways. In the present study, the silencing of *MnYorkie* markedly downregulated the transcription of *MnCactus* but upregulated the expression of six *AMPs*, namely, *MnALF1*, *MnALF2*, *MnALF3*, *MnCrus4*, *MnCrus5*, *MnCrus6*, and an immune-related gene *MnLyso1*. In addition, *MnYorkie* silencing *in vivo* decreased the susceptibility of prawns to Gram-positive and -negative bacterial infection. After *S. aureus* and *V. parahaemolyticus* challenge, the survival rate of prawns increased significantly after 2 days to 6 days, which corresponded to the period of *MnYorkie* knockdown. In *M. nipponense*, MnHippo inhibition promoted the translocation of MnYorkie into the nucleus and then regulated the transcription of *MnCactus*. The high MnCactus expression prevented the translocation of MnDorsal into the nucleus, thereby reducing the expression of some genes, including AMPs, to induce the antimicrobial state of the host.

In conclusion, a *Yorkie* gene and two *Cactus* isoforms (named *MnYorkie*, *MnCactus-a* and *MnCactus-b*) were identified from *M. nipponense*. *MnCactus-a* and *MnCactus-b* were formed by alternative splicing. Upon immune challenge with *S. aureus* and *V. parahaemolyticus*, the expression levels of *MnYorkie* and *MnCactus* mRNA were upregulated in the hemocytes and intestines of *M. nipponense*. RNAi experiments showed that *MnYorkie* knockdown affected the transcription of *MnCactus* and seven immune-related genes, the susceptibility of prawns to bacterial infections, and the survival rate of prawns. These results suggested that *MnYorkie* in the Hippo pathway might have remarkable biological roles in the immune defense of *M. nipponense* by negatively regulating the expression of several immune-related genes and promoting the transcription of *MnCactus*. Further studies are required to understand the exact mechanism of *MnYorkie* in crustaceans.

## Data Availability Statement

The original contributions presented in the study are included in the article/supplementary material. Further inquiries can be directed to the corresponding authors.

## Author Contributions

YH, data curation, formal analysis, funding acquisition, investigation, methodology, software, supervision, validation, visualization, writing - original draft, and writing - review and editing. QS, formal analysis, software, and visualization. JD, data curation, funding acquisition, and validation. QR, conceptualization, project administration, funding acquisition, resources, and writing - review and editing. All authors contributed to the article and approved the submitted version.

## Funding

The current study was supported by the National Natural Science Foundation of China (32002423), the Natural Science Foundation of Jiangsu Province (BK20180501), the Fundamental Research Funds for the Central Universities (B200202142), the Natural Science Fund of Colleges and universities in Jiangsu Province (14KJA240002), and Youth Support Project of Jiangsu Vocational College of Agriculture and Forestry (No. 2020kj011).

## Conflict of Interest

The authors declare that the research was conducted in the absence of any commercial or financial relationships that could be construed as a potential conflict of interest.

## Publisher’s Note

All claims expressed in this article are solely those of the authors and do not necessarily represent those of their affiliated organizations, or those of the publisher, the editors and the reviewers. Any product that may be evaluated in this article, or claim that may be made by its manufacturer, is not guaranteed or endorsed by the publisher.
